# What Factors Influence Symptom Reporting and Access to Healthcare During an Emerging Infectious Disease Outbreak? A Rapid Review of the Evidence

**DOI:** 10.1089/hs.2020.0126

**Published:** 2021-08-16

**Authors:** Patrice Carter, Odette Megnin-Viggars, G. James Rubin

**Affiliations:** Patrice Carter, PhD, and Odette Megnin-Viggars, PhD, are Senior Systematic Reviewers; both at the Centre for Outcomes Research and Effectiveness, Research Department of Clinical, Educational and Health Psychology, University College London, London, UK. Patrice Carter is also a Senior Systematic Reviewer, Health Economics & Outcomes Research Ltd, Cardiff, UK. G. James Rubin, PhD, is Assistant Director, Health Protection Unit in Emergency Preparedness and Response, Department of Psychological Medicine, Institute of Psychiatry, Psychology & Neuroscience, King's College London, UK.

**Keywords:** COVID-19, System reporting, Medical management/response, Infectious diseases

## Abstract

During any emerging infectious disease outbreak, people with symptoms of the illness are asked to report to a health service immediately to facilitate contact tracing. Several factors may influence a person's willingness to report symptoms and their ability to access healthcare services. Understanding these factors has become urgent during the COVID-19 pandemic. To determine which factors influence symptom reporting during an emerging infectious disease outbreak, we conducted a rapid review of the evidence. Studies included in the review were based on primary research, published in a peer-reviewed journal, written in English, included factors associated with symptom reporting or accessing healthcare, and were related to a major public health incident involving an infectious disease outbreak. Five themes were identified as facilitators of symptom reporting or accessing healthcare: accurate and informative communication about the disease and the need to seek help, symptom severity, concern about disease exposure, ease of access to healthcare facilities, and relationship with the healthcare provider. Seven themes were identified as barriers to symptom reporting or accessing healthcare: lack of knowledge of the disease and its treatment, fear of the disease and fear of subsequent treatments or requirements, stigmatization attached to having a disease, invasion of privacy, low concern about symptoms, economic consequences of disease diagnosis, and challenges related to attending a healthcare facility. For contract tracing services to be effective, members of the public need to have the capability, opportunity, and motivation to use them. The themes identified should be used to evaluate information provided to the public to ensure as many people as possible with relevant symptoms report them to a healthcare provider.

## Introduction

Governments around the world are using contact tracing services to provide a way out of lockdown during the coronavirus disease 2019 (COVID-19) pandemic. In the United Kingdom, the government has launched a test, trace, and isolate system. This requires people who develop a new and continuous cough, fever, or loss of their sense of taste or smell (the “index case”’) to follow a series of actions. First, they must schedule a test for COVID-19. Second, they (and any household members) must remain at home until they receive their test result. Third, for those who test positive, further isolation is required and a process of tracing the person's close contacts begins. Contacts are asked to quarantine themselves, and to request a test if they also develop symptoms. Early detection of people with COVID-19 helps prevents the spread the illness to others. A similar system applies to other infectious diseases such as Ebola virus disease, severe acute respiratory syndrome, and Middle East respiratory syndrome coronavirus.

Every step of this system relies on people adhering to guidance. To date, most attention has focused on the challenges involved in helping index cases or their contacts adhere to advice to remain in isolation or quarantine for the recommended period of time.^1^ In this article, we focus on the very first steps in the patient pathway: recognizing that one has relevant symptoms and reporting those symptoms to one's health service.

This process can itself be broken down into stages. Symptom perception is determined partly by physiological factors. These might include infection with COVID-19, but physical signs of illness can also be triggered by other medical or environmental factors. Symptom perception is influenced by multiple psychological and contextual factors, which are more likely to occur in people who expect to experience a given symptom or who are worried or anxious and are hence monitoring themselves for symptoms.^2,3^

Seeking help once a symptom has been perceived is then also determined by a range of factors. The person's attribution of their symptoms plays a key role; most symptoms experienced in everyday life are appraised as being transient and benign and are, therefore, not brought to the attention of the health services. This appraisal is based partly on the severity, suddenness, and duration of the symptom. Minor, gradually evolving, and transient symptoms are less likely to be interpreted as indicating something troubling.^4^ However, awareness of specific health risks is also important. For example, not recognizing a symptom as a possible warning sign is a key factor underlying delayed help-seeking for cancer^5,6^ and rheumatoid arthritis.^4,7,8^

Regardless of the cause of an individual's symptoms, once the person has decided they have a problem, the processes involved in the decision to access healthcare services in order to request a test or seek help can be complex and result in delay. For example, an analysis in England found that of the 1,732 people who described having what the authors call an “alarm” symptom for cancer over the past 3 months, 28.4% had not yet consulted a doctor about an unexplained lump, 30.4% had not sought help for unexplained pain, and 45.8% had not sought help for unexplained bleeding.^9^ Important factors influencing the decision to seek help include the fear of being seen as a “time-waster,”^4,5,10^ competing demands on one's time,^5^ worries about the impact or efficacy of any intervention that might be offered,^4,5^ pressure from friends and family,^11,12^ and perceptions that one is complying with official advice or using a service appropriately.^13-15^

We are not aware of any published systematic reviews that explore factors facilitating or deterring people from reporting symptoms during major health incidents involving an infectious disease outbreak. In this article, we report a rapid review of the literature to determine what factors influence symptom reporting during such an incident.

## Methods

Following the PRISMA (Preferred Reporting Items for Systematic Reviews and Meta-Analyses) guidelines,^16^ we developed a protocol for conducting a rapid review to determine what factors influence symptom reporting during major health incidents.

### Search Strategy

We developed a search strategy that included medical subject headings and free-text terms. Key words for the search included terms for epidemics and pandemics (eg, coronavirus, avian influenza, Ebola, Middle East respiratory syndrome, severe acute respiratory syndrome, swine flu) and terms characterizing symptom reporting (eg, help-seeking behavior, symptom reporting, stigma, requesting/asking for a test). Three electronic databases (Medline, PsycINFO, ProQuest) were searched for literature published from database inception to June 2020. The full list of search terms can be found in Appendix A (www.liebertpub.com/doi/suppl/10.1089/hs.2020.0126).

### Selection Criteria

For studies to be included in this review, they had to report on primary research; be published in a peer-reviewed journal; be written in English; include factors associated with reporting symptoms to a healthcare unit or accessing healthcare (ie, facilitators, barriers, or features associated with the reporting of symptoms or accessing of healthcare); and include participants with experience of a major health incident, which had to be viral, contagious, and not sexually transmitted. References from each database search were downloaded into EndNote and duplicates removed. Titles and abstracts of identified studies were screened by 2 reviewers for inclusion against criteria, until a good interrater reliability was observed (percentage agreement ≥90%). Initially, 10% of references were double-screened, and because interrater agreement was good (98%), the remaining references were screened by 1 reviewer. All primary-level studies included after the first screening of references were acquired in full and reevaluated for eligibility (see Appendix B for studies that were excluded during the full-text review: www.liebertpub.com/doi/suppl/10.1089/hs.2020.0126).

### Data Extraction and Synthesis

Data extraction and synthesis was conducted independently by 2 reviewers, and discrepancies or difficulties with coding were resolved through discussion. The quality of each study was assessed using the Critical Appraisals Skills Programme checklist for qualitative studies^17^ or the *BMJ* critical appraisal checklist for survey studies.^18^ A quality rating was assigned to each study, where ++ indicates that most (≥75%) or all of the checklist criteria have been met, + indicates that the checklist criteria have been partially met (≥50% to 75%), and – indicates that the majority of checklist criteria have not been met (<50%). Thematic synthesis was used to inductively identify emerging themes in qualitative findings across studies. Themes from individual studies were integrated into a wider context, and overarching categories of themes and subthemes were developed. Themes were derived from the data presented within the studies, and emerging themes were placed into a thematic map representing the relationship between the overarching categories, themes, and subthemes. Narrative synthesis was used to combine and summarize the findings from survey studies and was embedded within the context of the qualitative findings to provide an overall narrative of the data.

## Results

**Figure 1.**
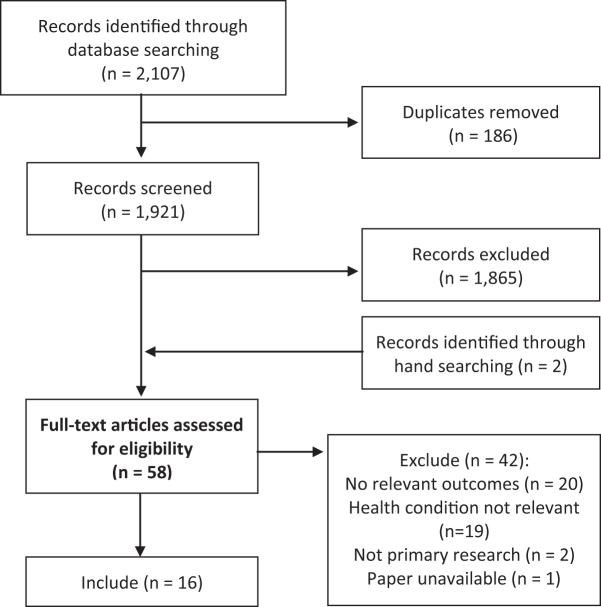
PRISMA flow chart of study search and selection process.

The systematic search of electronic databases generated a total of 2,107 references, and 2 additional references were identified through hand searching. Of these 2,109 references, 186 duplicates were removed. Of the remaining references, 1,865 articles did not meet the eligibility criteria of the review and were excluded based on either population, intervention, or study design. The remaining 58 articles were reviewed in full text. Sixteen studies met the eligibility criteria (see Figure 1 for flow chart of the study search and selection process).

### Included Studies

The characteristics of the included studies are summarized in the Supplemental Table (www.liebertpub.com/doi/suppl/10.1089/hs.2020.0126). Half of the studies used qualitative methods^11,19-25^ and half used quantitative surveys.^26-33^ The studies were conducted across 12 countries and included people who experienced the 2009 H1N1 influenza pandemic,^11,24,26,27,32^ 2014-2016 West African Ebola outbreak,^20,21,23,25,33^ avian influenza,^29,30^ acute respiratory illness,^22^ influenza-like illness,^28,31^ and any infectious disease.^19^

### Identified Themes

**Figure 2.**
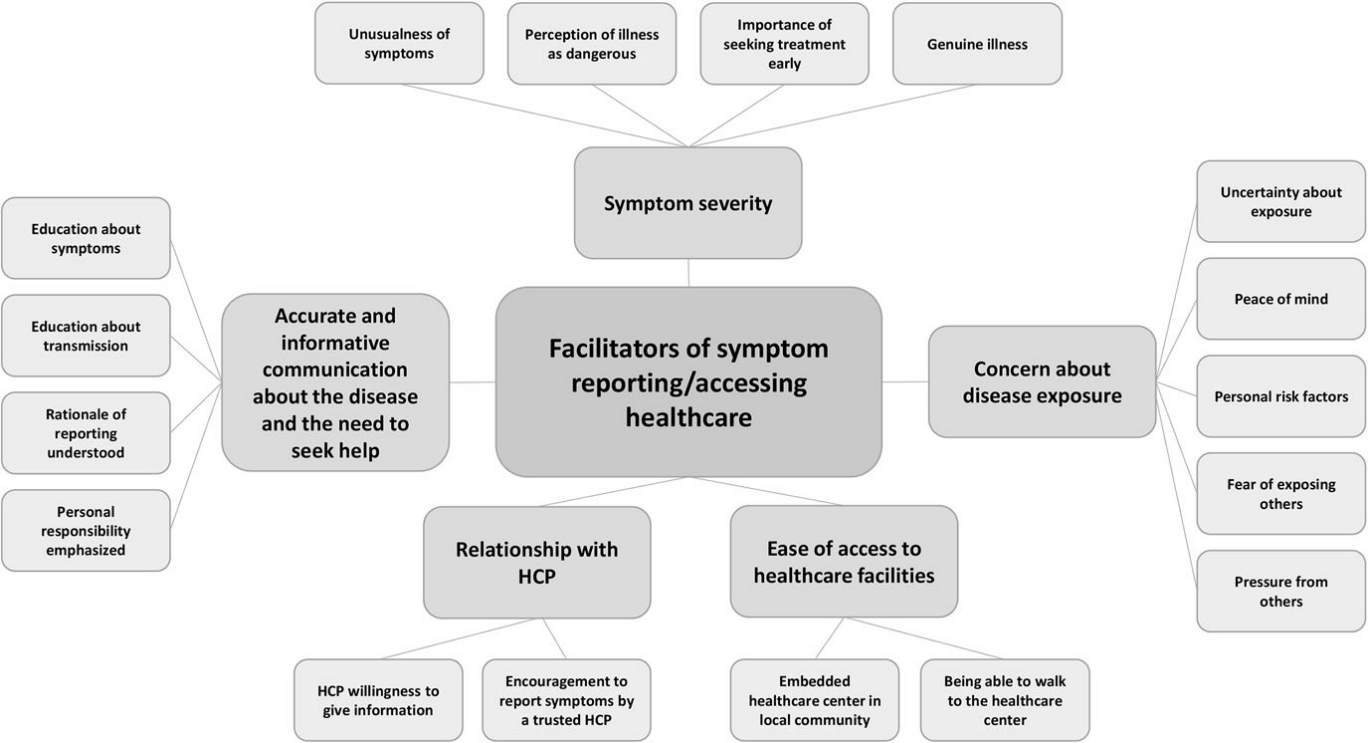
Theme map of facilitators for reporting symptoms or accessing healthcare. Abbreviation: HCP, healthcare provider.

**Figure 3.**
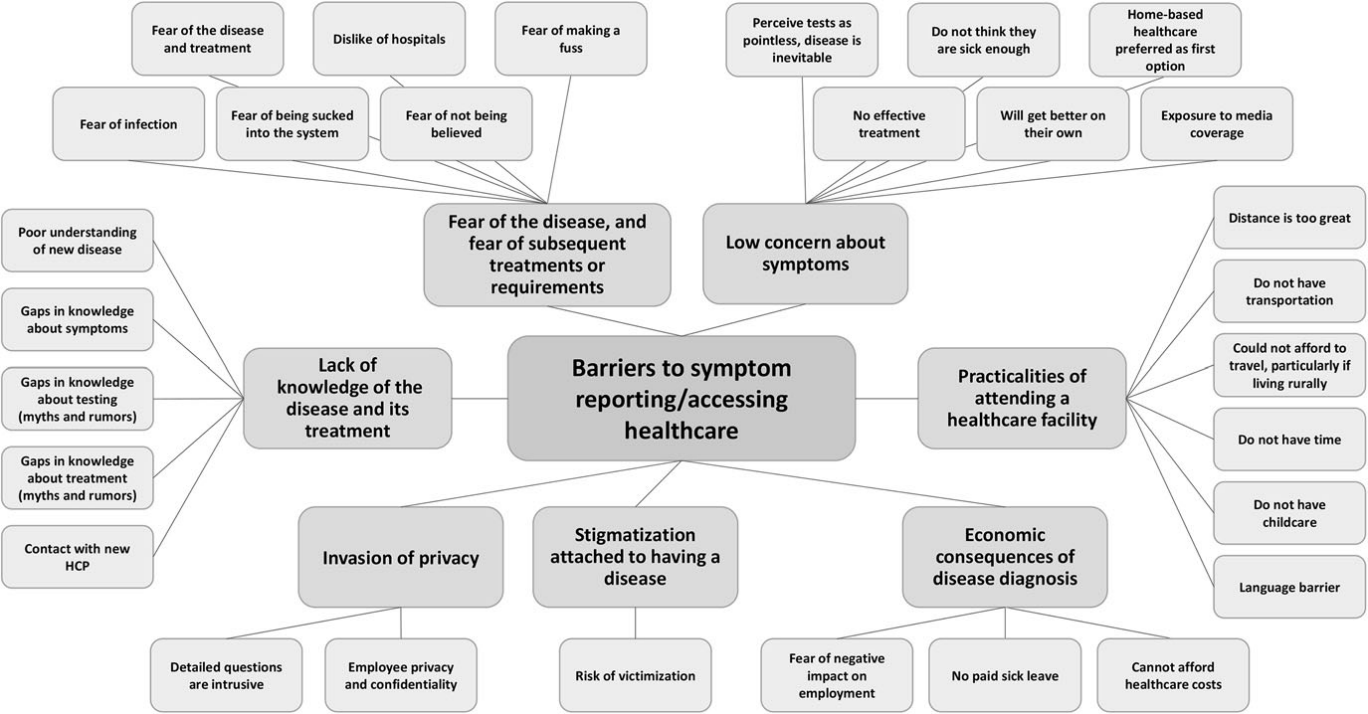
Theme map of barriers to reporting symptoms or accessing healthcare. Abbreviation: HCP, healthcare provider.

Five themes were identified as facilitators for reporting symptoms or accessing healthcare: accurate and informative communication about the disease and the need to seek help, symptom severity, concern about disease exposure, ease of access to healthcare facilities, and relationship with healthcare provider. Seven themes were identified as barriers to reporting symptoms or accessing healthcare: lack of knowledge of the disease and its treatment, fear of the disease and fear of subsequent treatment or requirements, stigmatization attached to having a disease, invasion of privacy, low concern about symptoms, economic consequences of disease diagnosis, and challenges related to attending a healthcare facility. See Figure 2 and Figure 3 for theme maps of facilitators and barriers to reporting symptoms. These themes (and subthemes) are elaborated in the following sections. The synthesis of quantitative survey data also revealed several demographic features associated with those who reported symptoms and accessed healthcare.

## Facilitators of Reporting Symptoms and Accessing Healthcare

### Accurate and Informative Communication

In 4 studies, participants in qualitative interviews and survey respondents both reported that education about symptoms^19,21,30,31^ and routes of transmission^30^ enabled them to feel confident about reporting symptoms and seeking help. The participants in these studies also emphasized they would be happier to report symptoms if they understood the rationale behind reporting, and if their personal responsibility was emphasized:

*I think most people would be happy if they knew there was a risk, if you could tell them why you're asking the questions.*^19^

Participants explained how being informed about the disease strengthened their resolve to seek treatment,^21^ and when people know that early access is key to getting treatment, they are more likely to visit healthcare facilities.^30^ Additionally, healthcare workers discussed that having knowledge is important to make people take action:

*If all the staff were educated on that, like if you've got D&V [diarrhea and vomiting], please report it; surely, they should take a little bit of ownership and report it.*^19^

Participants in this study also highlighted that having confidence that the reporting system was effective and efficient would facilitate reporting.^19^

### Symptom Severity

Participants in qualitative interviews and survey respondents of 2 studies^11,29^ reported that having unusual symptoms, particularly in terms of severity and duration, were powerful prompts to access healthcare:

*I've had a cold and I've had the flu and it only lasted about 2 days. But I had this for about a week and a half. It's the first I've had a cold that long and I was just thinking the worst.*^11^

Two surveys found that early access to healthcare was also facilitated when people perceived the illness as dangerous.^29,30^ Participants in qualitative interviews^21,29^ also reported being more motivated to seek treatment earlier when the importance of early response was emphasized, and where exposure to survivors built hope about recovery:

*We see people who come out [of the treatment center] and they are still alive … now we see that people can live.*^21^

Participants in 1 study expressed that if they felt confident that they were experiencing a genuine illness, they would feel comfortable about disclosing symptoms:

*If you're genuinely ill and you've got nothing to hide, then I'd be comfortable. It wouldn't bother me, I'd tell everything.*^19^

### Concern About Disease Exposure

One study found that participants reported being more motivated to seek advice if they were concerned or uncertain about exposure.^11^ Another related motivator was a desire to gain peace of mind:

*[I wanted] to confirm that I hadn't got this flu virus. So it was more so, you know, “you definitely haven't.” That was the reassurance I needed.*^11^

Furthermore, those with personal risk factors (including age and underlying health conditions) were particularly compelled to contact healthcare services.^11^ Social pressures also facilitated help-seeking behavior, both in terms of fears about exposing others to the disease and pressure from others to report symptoms.

### Ease of Access to Healthcare Facilities

The physical proximity of healthcare facilities and the importance that they were local and community-based were highlighted as facilitators to accessing healthcare because the people working in those facilities are more likely to be known and trusted.^20,25^ Being able to walk to facilities also resolved transportation issues from a practical perspective and provided an emotional facilitating factor as many participants across studies described an intense fear of ambulances.^20,22^

### Relationship with Healthcare Provider

One study found that participants reported being more likely to seek help if they were encouraged to do so by familiar and trusted healthcare professionals:

*My cousin works there … she has even gone to some houses to encourage them herself, because she knows them, they listen.*^20^

Help-seeking behavior was also facilitated through healthcare professionals' willingness to discuss symptoms and provide information.^22^ Conversely, having to contact a new and unknown healthcare provider was highlighted as a barrier that delayed the decision to seek healthcare.^24^

## Barriers to Reporting Symptoms and Accessing Healthcare

### Lack of Knowledge of the Disease and Its Treatment

Participants in 2 studies described a poor understanding of the characteristics of a new disease and how this could act as a barrier to help-seeking behavior.^19,25^ More specifically, the participants talked about gaps in knowledge of symptoms,^33^ gaps in knowledge about testing,^20,25^ and gaps in knowledge about treatment,^20,25^ and how these gaps could be filled with rumors and myths that could make people even more fearful about accessing healthcare:

*There were rumors that the needle to test you actually is poison and the test kills you.*^20^

*I hear they take the blood specimens of people but even there it is not clear to me. They can take the blood sample of somebody under the pretext that he/she has Ebola.*^25^

### Fear of the Disease and Treatments or Requirements

Fear emerged as a recurrent barrier to reporting symptoms, including fear of what may happen if a person catches the disease, fear of receiving treatment, and fear of the treatment system:

*They take the [the patient] away and that's it, you will never hear from them again.*^20^

Other fears included fear of being infected in a healthcare setting,^23^ fear of what happens after entering the health system after witnessing other sick people being taken away and never returning,^20,25^ fear of the hospital or clinic environment,^24,25,33^ and fear of the reaction they may receive from the healthcare worker, such as not being believed^19^ or being considered to make a fuss.^33^

### Stigmatization Attached to Having a Disease

In 3 studies on stigmatization, participants reported they anticipated stigmatization, and the desire to avoid it created a barrier to reporting symptoms.^19,22,23^ People worried about experiencing stigma in the workplace and in the wider community, and they were concerned about social stigma that may be attached to family members of those who had developed severe illness. One study discussed how respondents demonstrated social stigma toward caretakers, believing that people who did not take care of their families appropriately ended up with family members having severe respiratory cases.^22^

### Invasion of Privacy

Participants in a focus group reported they felt that detailed questioning about symptoms could be experienced as an invasion of privacy and create or increase feelings of anxiety and distrust.^19^ The need for protection of employee privacy and confidentiality was also raised, in the context of healthcare workers reporting symptoms.^19^

### Low Concern About Symptoms

Juxtaposed against the finding that concern about exposure could act as a facilitator to reporting symptoms, others experienced low concern about symptoms, which acted as a barrier to seeking help. For some people, this took the form of a fatalistic approach, as they perceived tests as pointless and disease inevitable^21^ or delayed the decision to seek healthcare due to the lack of an effective treatment.^24^ Others did not feel sufficiently ill to report symptoms or access healthcare.^25,29,33^

*Because based on what the medical people say, if it is high fever, then you will say this has gone beyond limit. Because there are times when we get malaria and common cold before this Ebola. So you cannot just start feeling feverish and you say you have to call 117.* ^25^

Some participants in qualitative interviews and survey respondents expressed the belief that there was no need to access healthcare because they would get better on their own,^22,29^ or they preferred to try home-based healthcare as a first-line treatment option.^23-25^

Studies also found that exposure to media coverage reduced worry and decreased healthcare service use.^32,25^

### Economic Consequences of Disease Diagnosis

Economic barriers to accessing healthcare were also experienced. Participants in 3 studies reported they could not afford healthcare,^23-25^ or they were worried about a negative impact on their employment or had no paid sick leave.^24^ One participant stated he could not afford to go to a healthcare center, and so he would pray.^25^

### Challenges Related to Attending a Healthcare Facility

For some people the healthcare facility was too far away,^29^ and a lack of transportation^23,24,29^ or not being able to afford to travel^29^ imposed practical barriers to accessing healthcare, particularly for those living in rural communities. Other barriers to help-seeking behavior included not having time to visit a healthcare facility,^29^ lacking childcare,^29^ and language or communication challenges.^24^

## Associated Features of Those Reporting Symptoms

Three studies found that women were more likely to report symptoms than men,^26,28,31^ but we are unable to determine if this is because women were more likely to contract the illness. Two studies reported that older people (at least 65 years of age) were less likely to report symptoms than those who could be categorized as younger or middle-aged, although these age bands were large, covering ages 18 to 49 years^26^ or ages 25 to 64 years.^28^ One study found that white respondents were more likely to report symptoms than Black respondents.^26^ However, the relationship between ethnicity and reporting is more complex, as the same study^26^ found that Native American/Alaska Native participants were more likely to report symptoms than white respondents. Finally, 2 studies found that people with higher levels of education were more likely to report symptoms than those with less education.^28,31^

## Associated Features of Those Seeking Healthcare

Two studies found that women were more likely to seek medical care than men.^26,31^ One study reported that those with primary-level education or lower were more likely to seek medical care than those with a tertiary level of education or higher.^31^ One study found that people from ethnic minority groups were more likely to access healthcare.^32^ Two studies reported that those with underlying health conditions^31,32^ were more likely to have sought medical care, and 1 of those studies found that people living in larger households were more likely to access healthcare.^32^ Again, within this review we cannot determine whether greater levels of health-seeking behavior by these groups is due to an increased likelihood that they contracted the illness. Finally, mixed results were reported across studies on the association between age and health-seeking behavior, with 2 studies finding that older adults (at least 60 to 65 years of age) were more likely to seek healthcare,^26,31^ and another study in which participants indicated they felt too old to seek care.^29^ Another 2 studies found no direct association between age and help-seeking behavior, but they found relationships with other variables. One study reported a relationship between age and gender, with male survey respondents under the age of 25 years being more likely to seek medical attention than female respondents under the age of 25 years.^27^ Another study reported a relationship between age and ethnicity—the longest duration between onset of symptoms and seeking initial treatment was found among an older age group of Black and minority ethnic respondents, whereas for white respondents the longest duration was reported by children and young people.^24^

## Discussion

### Principal Finding

The evidence within this review shows a number of distinct factors that can act as either facilitators or barriers to people reporting symptoms or seeking care from the health services during an emerging infectious disease outbreak. These factors include fear or concern about exposure to the disease, knowledge and information about the disease, the need to seek help, level of concern about symptoms, and practical considerations. Additional factors are related to invasion of privacy and stigmatization associated with having a disease. A number of demographic factors should also be considered including sex, age, ethnicity, and education.

### Interpretation of Results

Our thematic analysis identified 4 main themes that facilitate people either reporting symptoms or accessing healthcare facilities: symptom severity, concern about exposure, accurate and informative communication about the disease and its treatment, the need to seek help, the relationship with a healthcare provider, and ease of access. For example, when information is accurate and communicated effectively, people understand why they need to report symptoms and feel enabled to do so,^19^ whereas when people have a poor understanding of testing, the disease, or symptoms, they appear reluctant to visit a healthcare facility or report symptoms.^20,25^ Providing clear information about the disease, its treatment, and what happens when visiting a healthcare facility is shown to motivate and encourage people to attend facilities and report their symptoms. Public health officials need to ensure people receive appropriate information by working with the media who play an important role during major health crises.^34^ The call for clear communication is not new in the field of emergency response, but we make no apologies for raising it again. Without sustained effort on the part of official communicators, knowledge among the public can be surprisingly low. For example, 5 months into the COVID-19 pandemic, only 59% of the UK population knew what symptoms they should be alert to.^35^ The role of healthcare professionals is also important. Data show that people were more positive when healthcare providers were willing to give them information and talk to them.^22^ The information shared as well as the experiences of how people are regarded by healthcare professionals when attending a health facility provide positive feedback, which is fed into the community at large.

Accurate and informative communication about the disease and its treatment, and the need to seek help, are closely linked to the themes of “symptom severity” (a facilitator) and “low concern about symptoms” (a barrier). If people are not sufficiently educated about relevant symptoms, they may not access healthcare or report their symptoms. Clear advice on severity and the nature of symptoms is, therefore, needed. These data are consistent with previous research, which show that when people think their symptoms are minor or unimportant, they are less likely to report them.^4^ According to another study, when people believed they had a genuine illness, and that symptoms were severe or serious, they reported them.^19^ These findings support a study that found that the greater the perceived perception of illness severity, the more likely people are to engage in precautionary behaviors,^36^ such as contacting healthcare services. During the COVID-19 outbreak, a lack of reporting symptoms or seeking healthcare represents a substantial problem for testing and contact tracing services. Given that people are infectious even if asymptomatic, it is essential to encourage people to report even mild symptoms without delay. A focus on such messaging in communication efforts is required, as our data suggest that reporting mild symptoms runs contrary to most people's natural inclinations.

Anxiety about exposure also increased people's motivations to seek help or report symptoms. When people were concerned that their individual situation increased their susceptibility to a disease, they were more likely to engage with the healthcare system. In contrast, fear about what might happen next once they are admitted to the health system was identified consistently across studies, where it acted as a barrier to reporting symptoms. These findings suggest that reducing fear about being infected while visiting a healthcare setting or receiving treatment is important, while instilling a sufficiently realistic degree of concern about the illness that motivates people to engage with the system. Although we identified anxiety as a theme, the data showed little about other personal motivations for reporting symptoms or accessing healthcare. Further research exploring peoples belief systems and what personal motivations may drive them to report symptoms should be investigated

Practical issues related to logistics and cost were regularly reported in the evidence. Having a healthcare facility embedded in the local community and within walking distance was a facilitator for people reporting symptoms. However, if traveling to a facility was deemed too far, too expensive, or too difficult, it was seen as a barrier. The cost of accessing the healthcare system was also a consideration for many. In addition, indirect economic factors were important, such as people reporting difficulties with getting time off work and not having paid sick leave. During an emerging infectious disease outbreak, tackling economic barriers to seeking testing should be an essential component of any fully functioning public health response.

Evidence on the associated features of those seeking healthcare and those reporting symptoms showed clear differences between genders, age groups, and ethnic backgrounds. Studies showed that women consistently reported symptoms more often and were more likely to seek healthcare than men, highlighting that more needs to be done to engage the male population. The data were not consistent in relation to age or ethnicity, however.

### Strengths and Limitations

Despite this being a rapid review of evidence, we conducted a comprehensive search strategy across multiple databases, with screening conducted by 2 independent reviewers. The review included 16 studies, providing a depth of evidence from across the globe and supporting the generalizability of findings. We described the studies in detail and demonstrated clear themes in the analysis to enhance understanding of the existing evidence. However, as with all rapid reviews, several limitations must be considered. We cannot rule out the possibility that other potentially relevant evidence exists that has not been included. Additionally, several studies included in our review were rated moderate or low quality, which was often due to suboptimal reporting of participants and research methods.

## Recommendations

Findings from this review suggest that in the context of the COVID-19 pandemic, governments seeking to increase the proportion of symptomatic people who access testing services should:

- Ensure information on symptoms is clear and that reporting a new and continuous cough, a fever, or a loss of sense of taste or smell is important. It should be emphasized that even mild or minor symptoms must be reported without delay.
- Make testing facilities easy to access and remove any economic barriers associated with their use.
- Provide assurance that testing facilities are safe from infection.
- Target the male population to increase their uptake of services.

## Supplementary Material

Supplemental data

Supplemental data

Supplemental data
