# Supplementary material for: What Factors Influence Symptom Reporting and Access to Healthcare During an Emerging Infectious Disease Outbreak? A Rapid Review of the Evidence
Source: Health Secur. 2021 Aug 16;19(4):353–63. doi: 10.1089/hs.2020.0126 (PMC8403196; doi:10.1089/hs.2020.0126)
Supplement: Supplemental data [file Supp_App1.docx]

**Appendix A**

Ovid MEDLINE(R) and Epub Ahead of Print, In-Process & Other Non-Indexed Citations and Daily 1946 to June 09, 2020

Date searched: 10^th^ June 2020

| # | Search terms | Hits |
| --- | --- | --- |
| 1 | Pandemics/ | 9950 |
| 2 | Coronavirus Infections/ | 9674 |
| 3 | Disease outbreaks/ | 79399 |
| 4 | "avian influenza".mp. | 10791 |
| 5 | Influenza A Virus, H1N1 Subtype/ or "bird flu".mp. or Influenza A Virus, H5N1 Subtype/ | 20603 |
| 6 | Coronavirus/ or coronavirus.mp. or covid 19.mp. or covid-19.mp. | 34377 |
| 7 | Ebola.mp. | 9045 |
| 8 | "equine influenza".mp. | 787 |
| 9 | H1N1.mp. | 21000 |
| 10 | H5N1.mp. | 7902 |
| 11 | MERs.mp. or Middle East Respiratory Syndrome Coronavirus/ | 4868 |
| 12 | "Middle East respiratory syndrome".mp. | 2325 |
| 13 | pandemic*.mp. | 37034 |
| 14 | Epidemics/ or epidemic*.mp. | 109445 |
| 15 | SARS Virus/ or SARs.mp. or Severe Acute Respiratory Syndrome/ | 16796 |
| 16 | "severe acute respiratory syndrome".mp. | 12771 |
| 17 | "swine flu".mp. | 956 |
| 18 | Help-Seeking Behavior/ | 745 |
| 19 | Help-seek*.mp. | 6215 |
| 20 | (symptom* adj3 report*).mp. | 41088 |
| 21 | (request* or ask* or demand* or call or contact* or access* or inquire* or enquire* or consult* or seek* or requir* or appl* or order* or need* or want*) adj3 (test*) | 94989 |
| 22 | (test* or diagnos*) adj3 (stigma* or fear* or worr* or shame* or reluctan* or knowledge or recogni* or risk perception or perceived risk) | 26496 |
| 23 | Or/1-17 | 248141 |
| 24 | Or/18-22 | 167232 |
| 25 | 23 and 24 | 2169 |
| 26 | limit 25 to humans | 1542 |
| 27 | limit 26 to english language | 1418 |

APA PsycInfo 1806 to June Week 1 2020

Date searched: 10^th^ June 2020

| # | Search terms | Hits |
| --- | --- | --- |
| 1 | exp Pandemics/ | 534 |
| 2 | "avian influenza".mp. | 114 |
| 3 | "bird flu".mp. | 34 |
| 4 | coronavirus.mp. or covid 19.mp. or covid-19.mp. | 280 |
| 5 | Ebola.mp. | 414 |
| 6 | "equine influenza".mp. | 0 |
| 7 | H1N1.mp. | 495 |
| 8 | H5N1.mp. | 57 |
| 9 | MERs.mp. or "Middle East respiratory syndrome".mp. | 99 |
| 10 | pandemic*.mp. | 2015 |
| 11 | epidemic.mp. or exp Epidemics/ | 12570 |
| 12 | SARs.mp. or "severe acute respiratory syndrome".mp. | 525 |
| 13 | "swine flu".mp. | 92 |
| 14 | exp Help Seeking Behavior/ | 13495 |
| 15 | Help-seek*.mp. | 10538 |
| 16 | (symptom* adj3 report*).mp. | 19817 |
| 17 | (request* or ask* or demand* or call or contact* or access* or inquire* or enquire* or consult* or seek* or requir* or appl* or order* or need* or want*) adj3 (test*) | 25925 |
| 18 | (test* or diagnos*) adj3 (stigma* or fear* or worr* or shame* or reluctan* or knowledge or recogni* or risk perception or perceived risk) | 22000 |
| 19 | Or/1-13 | 14733 |
| 20 | Or/14-18 | 84131 |
| 21 | 19 and 20 | 267 |
| 22 | limit 21 to humans | 257 |
| 23 | limit 22 to english language | 253 |

ProQuest (Coronavirus Research Database, Public Health Database, Social Science Database, Sociology Database and Internal Bibliography of the Social Science [IBSS])

Date searched: 11^th^ June 2020

| # | Search terms | Hits |
| --- | --- | --- |
| 1 | "avian influenza" or "bird flu” or Coronavirus or “covid 19” or covid-19 or Ebola or "equine influenza" or H1N1 or H5N1 or MERs or "Middle East respiratory syndrome" or pandemic* or epidemic* or SARs or "severe acute respiratory syndrome" or "swine flu" |  |
| 2 | Help-seek* or (symptom* N/3 report*) or (request* N/3 test*) or (ask* N/3 test*) or (demand* N/3 test*) or (call N/3 test*) or (contact* N/3 test*) or (access* N/3 test*) or (inquire* N/3 test*) or (enquire* N/3 test*) or (consult* N/3 test*) or (seek* N/3 test*) or (requir* N/3 test*) or (appl* N/3 test*) or (order* N/3 test*) or (need* N/3 test*) or (want* N/3 test*) or (test* N/3 stigma*) or (test* N/3 fear*) or (test* N/3 worr*) or (test* N/3 shame*) or (test* N/3 reluctan*) or (test* N/3 knowledge) or (test* N/3 recogni*) or (test* N3 “risk perception”) or (test* N/3 “perceived risk”) or (diagnos* N/3 stigma*) or (diagnos* N/3 fear*) or (diagnos* N/3 worr*) or (diagnos* N/3 shame*) or (diagnos* N/3 reluctan*) or (diagnos* N/3 knowledge) or (diagnos* N/3 recogni*) or (diagnos* N3 “risk perception”) or (diagnos* N/3 “perceived risk”) |  |
| 3 | Search anywhere except full-text |  |
| 4 | limit to peer reviewed; Scholarly journals; Article; English language | 679 |
