# Supplementary material for: What Factors Influence Symptom Reporting and Access to Healthcare During an Emerging Infectious Disease Outbreak? A Rapid Review of the Evidence
Source: Health Secur. 2021 Aug 16;19(4):353–63. doi: 10.1089/hs.2020.0126 (PMC8403196; doi:10.1089/hs.2020.0126)
Supplement: Supplemental data [file Supp_App2.docx]

**Appendix B**

| **Study ID** | **Reason for Exclusion** | **Bibliographic reference** |
| --- | --- | --- |
| Agarwal 2014 | No relevant outcomes | Agarwal, V. (2014). A/H1N1 vaccine intentions in college students: An application of the theory of planned behavior. Journal of American College Health, 62(6), 416-424. |
| Almutairi 2015 | No relevant outcomes | Almutairi, K. M., Al Helih, E. M., Moussa, M., Boshaiqah, A. E., Saleh Alajilan, A., Vinluan, J. M., & Almutairi, A. (2015). Awareness, attitudes, and practices related to coronavirus pandemic among public in Saudi Arabia. Family & community health, 38(4), 332-340. |
| Arendt 2013 | Health condition not relevant | Arendt, S., Rajagopal, L., Strohbehn, C., Stokes, N., Meyer, J., & Mandernach, S. (2013). Reporting of foodborne illness by US consumers and healthcare professionals. International journal of environmental research and public health, 10(8), 3684-3714. |
| Atiglo 2018 | Health condition not relevant | Atiglo, D. Y., Larbi, R. T., Kushitor, M. K., Biney, A. A., Asante, P. Y., Dodoo, N. D., & Dodoo, F. N. A. (2018). Sense of community and willingness to support malaria intervention programme in urban poor Accra, Ghana. Malaria journal, 17(1), 289. |
| Balkhy 2010 | No relevant outcomes | Balkhy, H. H., Abolfotouh, M. A., Al-Hathlool, R. H., & Al-Jumah, M. A. (2010). Awareness, attitudes, and practices related to the swine influenza pandemic among the Saudi public. BMC infectious diseases, 10(1), 42. |
| Dodds 2020 | Not primary research | Dodds, C., & Fakoya, I. (2020). Covid-19: ensuring equality of access to testing for ethnic minorities. BMJ, 369. |
| Donovan 2001 | Health condition not relevant | Donovan, B., Knight, V., McNulty, A. M., Wynne‐Markham, V., & Kidd, M. R. (2001). Gonorrhoea screening in general practice: perceived barriers and strategies to improve screening rates. Medical journal of Australia, 175(8), 412-414. |
| Furr-Holden 2020 | Not primary research | Furr-Holden, D., Carter-Pokras, O., Kimmel, M., & Mouton, C. (2020). Access to care during a global health crisis. Health Equity, 4(1), 150-157. |
| Ho 2015 | Health condition not relevant | Ho, V., Zainal, N. H., Lim, L., Ng, A., Silva, E., & Kandiah, N. (2015). Voluntary cognitive screening: characteristics of participants in an Asian setting. Clinical interventions in aging, 10, 771. |
| Hui 2013 | Health condition not relevant | Hui, C. L., Tang, J. Y., Wong, G. H., Chang, W. C., Chan, S. K., Lee, E. H., & Chen, E. Y. (2013). Predictors of help-seeking duration in adult-onset psychosis in Hong Kong. Social psychiatry and psychiatric epidemiology, 48(11), 1819-1828. |
| Kfouri 2013 | No relevant outcomes | Kfouri, R. D. Á., & Richtmann, R. (2013). Influenza vaccine in pregnant women: immunization coverage and associated factors. Einstein (Sao Paulo), 11(1), 53-57. |
| Langeni 2007 | Health condition not relevant | Langeni, T. (2007). Contextual factors associated with treatment-seeking and higher-risk sexual behaviour in Botswana among men with symptoms of sexually transmitted infections. African Journal of AIDS Research, 6(3), 261-269. |
| Lau 2005 | No relevant outcomes | Lau, J. T., Yang, X., Tsui, H. Y., & Kim, J. H. (2005). Impacts of SARS on health-seeking behaviors in general population in Hong Kong. Preventive medicine, 41(2), 454-462. |
| Lester 2016 | No relevant outcomes | Lester, J., Paige, S., Chapman, C. A., Gibson, M., Jones, J. H., Switzer, W. M., ... & Frost, S. D. (2016). Assessing commitment and reporting fidelity to a text message-based participatory surveillance in rural western Uganda. PloS one, 11(6). |
| Lugova 2017 | Health condition not relevant | Lugova, H., & Wallis, S. (2017). Cross-sectional survey on the dengue knowledge, attitudes and preventive practices among students and staff of a public university in Malaysia. Journal of community health, 42(2), 413-420. |
| Nielsen 2016 | Health condition not relevant | Nielsen, A., Marrone, G., & De Costa, A. (2016). Chlamydia trachomatis among youth-testing behaviour and incidence of repeat testing in Stockholm County, Sweden 2010-2012. PloS one, 11(9). |
| Olowokure 2012 | No relevant outcomes | Olowokure, B., Odedere, O., Elliot, A. J., Awofisayo, A., Smit, E., Fleming, A., & Osman, H. (2012). Volume of print media coverage and diagnostic testing for influenza A (H1N1) pdm09 virus during the early phase of the 2009 pandemic. Journal of clinical virology, 55(1), 75-78. |
| Orton 2006 | Health condition not relevant | Orton, S. L., Stramer, S. L., & Dodd, R. Y. (2006). Self‐reported symptoms associated with West Nile virus infection in RNA‐positive blood donors. Transfusion, 46(2), 272-277. |
| Pearson 2008 | Health condition not relevant | Pearson, S., & Makadzange, P. (2008). Help‐seeking behaviour for sexual‐health concerns: a qualitative study of men in Zimbabwe. Culture, Health & Sexuality, 10(4), 361-376. |
| Perez 2012 | No relevant outcomes | Perez, V., Uddin, M., Galea, S., Monto, A. S., & Aiello, A. E. (2012). Stress, adherence to preventive measures for reducing influenza transmission and influenza-like illness. J Epidemiol Community Health, 66(7), 605-610. |
| Philen 1989 | No relevant outcomes | Philen, R., Mckinley, T., Kilbourne, E., & Parrish, R. G. (1989). Mass sociogenic illness by proxy: parentally reported epidemic in an elementary school. The Lancet, 334(8676), 1372-1376. |
| Plant 2010 | Health condition not relevant | Plant, A., Montoya, J. A., Rotblatt, H., Kerndt, P. R., Mall, K. L., Pappas, L. G., ... & Klausner, J. D. (2010). Stop the sores: the making and evaluation of a successful social marketing campaign. Health Promotion Practice, 11(1), 23-33. |
| Plant 2014 | Health condition not relevant | Plant, A., Javanbakht, M., Montoya, J. A., Rotblatt, H., O’Leary, C., & Kerndt, P. R. (2014). Check Yourself: A social marketing campaign to increase syphilis screening in Los Angeles County. Sexually transmitted diseases, 41(1), 50-57. |
| Randle 2018 | Health condition not relevant | Randle, J., Nelder, M., Sider, D., & Hohenadel, K. (2018). Characterizing the health and information-seeking behaviours of Ontarians in response to the Zika virus outbreak. Canadian Journal of Public Health, 109(1), 99-107. |
| Rodriguez-Valero 2018 | Health condition not relevant | Rodriguez-Valero, N., Oroz, M. L., Sanchez, D. C., Vladimirov, A., Espriu, M., Vera, I., ... & Carbayo, M. J. L. (2018). Mobile based surveillance platform for detecting Zika virus among Spanish Delegates attending the Rio de Janeiro Olympic Games. PloS one, 13(8). |
| Saak 2018 | No relevant outcomes | Saak, A. E., & Hennessy, D. A. (2018). A model of reporting and controlling outbreaks by public health agencies. Economic Theory, 66(1), 21-64. |
| Shen 2020 | No relevant outcomes | Shen, C., Chen, A., Luo, C., Zhang, J., Feng, B., & Liao, W. (2020). Using Reports of Symptoms and Diagnoses on Social Media to Predict COVID-19 Case Counts in Mainland China: Observational Infoveillance Study. Journal of Medical Internet Research, 22(5), e19421. |
| Stein 2014 | No relevant outcomes | Stein, M. L., Van Steenbergen, J. E., Chanyasanha, C., Tipayamongkholgul, M., Buskens, V., van der Heijden, P. G., ... & Kretzschmar, M. E. (2014). Online respondent-driven sampling for studying contact patterns relevant for the spread of close-contact pathogens: a pilot study in Thailand. PloS one, 9(1). |
| Stein 2015 | No relevant outcomes | Stein, M. L., van der Heijden, P. G., Buskens, V., van Steenbergen, J. E., Bengtsson, L., Koppeschaar, C. E., ... & Kretzschmar, M. E. (2015). Tracking social contact networks with online respondent-driven detection: who recruits whom?. BMC infectious diseases, 15(1), 522. |
| Stewart 2015 | No relevant outcomes | Stewart Jr, W. H., May, R. C., & Ledgerwood, D. E. (2015). Do you know what I know? Intent to share knowledge in the US and Ukraine. Management International Review, 55(6), 737-773. |
| Stockwell 2017 | No relevant outcomes | Stockwell, M. S., Marchant, C. D., Wodi, A. P., Barnett, E. D., Broder, K. R., Jakob, K., ... & Sharma, D. (2017). A multi-site feasibility study to assess fever and wheezing in children after influenza vaccines using text messaging. Vaccine, 35(50), 6941-6948. |
| Tan 2017 | Health condition not relevant | Tan, Q., Hildon, Z. J., Singh, S., Jing, J., Thein, T. L., Coker, R., ... & Leo, Y. S. (2017). Comparing patient and healthcare worker experiences during a dengue outbreak in Singapore: understanding the patient journey and the introduction of a point-of-care test (POCT) toward better care delivery. BMC infectious diseases, 17(1), 503. |
| Tengbeh 2018 | No relevant outcomes | Tengbeh, A. F., Enria, L., Smout, E., Mooney, T., Callaghan, M., Ishola, D., ... & Lees, S. (2018). “We are the heroes because we are ready to die for this country”: Participants' decision-making and grounded ethics in an Ebola vaccine clinical trial. Social Science & Medicine, 203, 35-42. |
| Thomson 2019 | Health condition not relevant | Thomson, R. M., Henderson, H. J., & Smith-Palmer, A. (2019). An outbreak of Salmonella Saintpaul in a Scottish childcare facility: the influence of parental under-reporting. BMC infectious diseases, 19(1), 847. |
| Tolia 2020 | No relevant outcomes | Tolia, V. M., Chan, T. C., & Castillo, E. M. (2020). Preliminary Results of Initial Testing for Coronavirus (COVID-19) in the Emergency Department. Western Journal of Emergency Medicine, 21(3), 503. |
| van den Hof 2001 | No relevant outcomes | van den Hof, S., Meffre, C. M., Conyn-van Spaendonck, M. A., Woonink, F., de Melker, H. E., & van Binnendijk, R. S. (2001). Measles outbreak in a community with very low vaccine coverage, the Netherlands. Emerging infectious diseases, 7(3 Suppl), 593. |
| Vasconcelos 1999 | Health condition not relevant | da C Vasconcelos, P. F., Lima, J. W., Raposo, M. L., Rodrigues, S. G., Rosa, J. F., Amorim, S. M., ... & Rosa, A. P. (1999). Seroepidemiologic survey in Sao Luis Island, State of Maranhao, Brazil, during a dengue fever epidemics. Revista da Sociedade Brasileira de Medicina Tropical, 32(2). |
| Viveki 2012 | Paper unavailable | Viveki, R. G., Halappanavar, A. B., Patil, M. S., Joshi, A. V., Gunagi, P., & Halki, S. B. (2012). Swine flu (H1N1 influenza): awareness profile of visitors of swine flu screening booths in Belgaum city, Karnataka. Journal of the Indian Medical Association, 110(6), 358-361. |
| Wang 2015 | Health condition not relevant | Wang, Q., Brenner, S., Leppert, G., Banda, T. H., Kalmus, O., & De Allegri, M. (2015). Health seeking behaviour and the related household out-of-pocket expenditure for chronic non-communicable diseases in rural Malawi. Health policy and planning, 30(2), 242-252. |
| Yang 2012 | No relevant outcomes | Yang, Z. J. (2012). Too scared or too capable? Why do college students stay away from the H1N1 vaccine?. Risk Analysis: An International Journal, 32(10), 1703-1716. |
| Yom-Tov 2015 | No relevant outcomes | Yom‐Tov, E., Johansson‐Cox, I., Lampos, V., & Hayward, A. C. (2015). Estimating the secondary attack rate and serial interval of influenza‐like illnesses using social media. Influenza and other respiratory viruses, 9(4), 191-199. |
| Zhang 2009 | Health condition not relevant | Zhang, Q., Lauderdale, D., Mou, S., Parish, W. I., Laumann, E. O., & Schneider, J. (2009). Socioeconomic disparity in healthcare-seeking behavior among Chinese women with genitourinary symptoms. Journal of Women's Health, 18(11), 1833-1839. |
