# Supplementary material for: What Factors Influence Symptom Reporting and Access to Healthcare During an Emerging Infectious Disease Outbreak? A Rapid Review of the Evidence
Source: Health Secur. 2021 Aug 16;19(4):353–63. doi: 10.1089/hs.2020.0126 (PMC8403196; doi:10.1089/hs.2020.0126)
Supplement: Supplemental data [file Supp_Table1.docx]

Supplemental Table. Characteristics of Included Studies

| *Author and Country* | *Study Design* | *Infectious Disease* | *Participants* | *Study Aim* | *Sample Size* | *Percentage Female* | *Percentage Black and Minority Ethnic (Non-White)* | *Included Themes* | *Study Quality^a^* |
| --- | --- | --- | --- | --- | --- | --- | --- | --- | --- |
| **Qualitative Studies** | | | | | | | | | |
| Aghaizu 2011^19^  England, Germany, Greece, Hungary | Qualitative: focus groups | Any infectious disease | Hospital-based healthcare workers | Determine healthcare workers’ attitudes to monitoring their sickness, absence, and reporting infectious disease symptoms | 49 | NR | NR | - Facilitators of symptom reporting:   - Accurate and informative communication about the disease and the need to seek help   - Symptom severity - Barriers to symptom reporting:   - Fear of the disease and fear of subsequent treatments or requirements   - Lack of knowledge of the disease and its treatment   - Invasion of privacy   - Economic consequences of disease diagnosis   - Stigmatization attached to having a disease | + |
| Carter 2017a^20^  Sierra Leone | Qualitative: in-person interview | Ebola | Adults living in Ebola-affected communities | Determine barriers and enablers of treatment seeking behavior for Ebola | 35 | NR | NR | - Facilitators of symptom reporting:   - Ease of access   - Relationship with healthcare provider - Barriers to symptom reporting:   - Fear of the disease and fear of subsequent treatments or requirements   - Lack of knowledge of the disease and its treatment | + |
| Carter 2017b^21^  Sierra Leone | Qualitative: in-person interviews, focus groups, and questionnaires | Ebola | Adults living in Ebola-affected communities | Determine barriers and enablers of treatment-seeking behavior for Ebola | 350 | NR | NR | - Facilitators of symptom reporting:   - Accurate and informative communication about the disease and the need to seek help   - Relationship with healthcare provider - Barriers to symptom reporting   - Low concern about symptoms | + |
| Lohiniva 2011^22^  Egypt | Qualitative: focus groups and semistructured interviews | Acute respiratory illness (ARI) | Household caregiver (defined as the primary caretaker of children or other household members during ARI episodes) | Explore the classification of ARI, to understand modes of transmission, symptoms of severity, and to explore health-seeking behaviors and home management practices | 18 focus groups and 20 interviews | 100 | NR | - Facilitators of symptom reporting:   - Ease of access   - Relationship with healthcare provider - Barriers to symptom reporting   - Low concern about symptoms   - Stigmatization attached to having a disease | + |
| McLean 2018^23^  Liberia | Qualitative: semistructured interviews | Ebola | Individuals from households who reported being sick | Explore experiences of illness, impact of disease, and mortality | 226 | 55 | NR | - Barriers to symptom reporting:   - Practicalities of attending healthcare facility   - Economic consequences of disease diagnosis   - Fear of the disease and fear of subsequent treatments of requirements   - Stigmatization attached to having a disease   - Low concern about symptoms | - |
| Rubin 2010a^11^  UK | Qualitative: semistructured interviews | Influenza A (H1N1) | General adult population | Understand why people contacted the health service about swine flu (H1N1) and to understand patients’ motivations for seeking advice | 5,419 | 58 | 27 | - Facilitators of symptom reporting:   - Symptom severity   - Concern about disease exposure | ++ |
| Soyemi 2009^24^  US | Qualitative: telephone interviews | Influenza (pH1N1) | People from Illinois who had been hospitalized for pH1N1 | Identify individual-level factors relating to health-seeking behavior | 2,824 data surveillance records and 33 interviews | 53 surveillance records and 42 interviews | 55 | - Barriers to symptom reporting:   - Practicalities of attending healthcare facility   - Economic consequences of disease diagnosis   - Fear of the disease, and fear of subsequent treatments or requirements   - Stigmatization attached to having a disease   - Low concern about symptoms - Associated features of those reporting symptoms:   - Demographics | + |
| Yamanis 2016^25^  Sierra Leone | Qualitative: semistructured interviews | Ebola | Adults living in Ebola-affected communities | Explore perceptions and intentions of those using the Ebola response system | 30 | 47 | NR | - Facilitators of symptom reporting:   - Ease of access - Barriers to symptom reporting:   - Fear of the disease, and fear of subsequent treatments or requirements   - Low concern about symptoms   - Lack of knowledge of the disease and its treatment   - Economic consequences of disease diagnosis | ++ |
| **Quantitative Studies** | | | | | | | | | |
| Biggerstaff 2012^26^  US | Quantitative: telephone survey | H1N1 influenza-like illness (ILI) | General adult population reporting ILI symptoms | Characterize people reporting ILI and to evaluate trends in healthcare-seeking behaviors, clinical diagnosis, and treatment of pH1N1 | 216,431 | 62 | 22 | - Associated features of reporting symptoms - Demographics | + |
| Brooks-Pollock 2011^27^  England | Quantitative: online survey | H1N1 influenza-like illness (ILI) | General adult population reporting ILI symptoms | Quantify healthcare-seeking behavior during the 2009 H1N1v epidemic | 1,522 | NR | NR | - Associated features of reporting symptoms - Demographics | + |
| Kjelso 2016^28^  Denmark | Quantitative: online data from “Influmeter” website | Influenza-like illness (ILI) | General adult population reporting ILI symptoms | Describe the participants representatively using a new online system (compared with the Danish population) and to compare the signal detection to existing surveillance systems | 1,089 | 67 | NR | - Associated features of those reporting symptoms - Demographics | - |
| Kreslake 2016^29^  Indonesia | Quantitative: healthcare utilization survey and in-person survey | Avian influenza (HPAI H5N1) | General adult population reporting ILI symptoms (survey data). Interviews conducted among those with and without symptoms | Determine the use of healthcare facilities, perceptions of healthcare services, and intentions to seek treatment in hypothetical scenarios | 2,520 | NR | NR | - Facilitators of symptom reporting   - Symptom severity - Barriers to symptom reporting   - Low concern about symptoms   - Economic consequences of disease diagnosis   - Practicalities of attending healthcare facility   - Stigmatization attached to having a disease - Associated features of those seeking healthcare   - Demographics | - |
| Manabe 2012^30^  Vietnam | Quantitative: in-person interviews | Avian influenza (H5H1) | General population of those living in affected and non-affected communities | Assess knowledge, attitudes, practice, and emotional response to H5N1 | 543 | 68 | NR | - Facilitators of symptom reporting   - Symptom severity   - Accurate and informative communication about the disease and the need to seek help | + |
| Meng 2016^31^  Hong Kong | Quantitative: telephone surveys | Influenza-like illness (ILI) | General adult population | Compare healthcare-seeking behaviors and self-medication of ILI patients between summer and winter influenza epidemics | 1,040 | 62 | NR | - Facilitators of symptom reporting   - Accurate and informative communication about the disease and the need to seek help - Associated features of those reporting symptoms   - Demographics | + |
| Rubin 2010b^32^  UK | Quantitative: telephone survey | Influenza A (H1N1) | General adult population | Determine associations between worry about catching swine flu (H1N1) and level of media coverage, and the role of media coverage and advertising in predicting accessing healthcare | 33 | 59 | 7 | - Barriers to symptom reporting   - Low concern about symptoms   - Concern about disease exposure - Associated features of those reporting symptoms   - Demographics | + |
| Tate 2017^33^  US | Quantitative: electronic or mailed survey | Ebola | People from New York City who had been part of the active monitoring scheme for Ebola | Evaluate frequency of reporting false data during active Ebola monitoring, factors associated with reporting false data, and the psychosocial impact of preferences during the Ebola active monitoring | 393 | 41 | NR | - Barriers to symptom reporting:   - Fear of the disease and fear of subsequent treatments or requirements   - Low concern about symptoms   - Lack of knowledge of the disease and its treatment | + |

^a^Study quality: Assessed using relevant tool: Critical Appraisal Skills Programme checklist for qualitative studies and BMJ survey checklist for quantitative surveys. Scores: ++, most of checklist criteria met; +, some of checklist criteria met; -, insufficient checklist criteria met.

Abbreviations: ARI, acute respiratory illness; ILI, influenza-like illness; H1N1, influenza A; NR, not reported.
